# Pre-treatments modulate drying characteristics, moisture removals, pigments and browning substrates, flavor, and microstructure in hot air dried daylily (*Hemerocallis citrina*)

**DOI:** 10.3389/fnut.2026.1896965

**Published:** 2026-08-04

**Authors:** Yongkang Xie, Junjun Geng, Xuanxuan Yang, Yan Zhao, Liuyan Wang

**Affiliations:** Agricultural Product Processing Research Center, Henan Academy of Agricultural Sciences, Zhengzhou, China

**Keywords:** cellular integrity, color preserving, daylilies, flavor, moisture removal

## Abstract

**Introduction:**

Hot air drying (HAD) of daylilies frequently results in prolonged drying. Severe browning, pigment degradation, and unacceptable colchicine retention, compromising both safety and market value. Selecting an appropriate pretreatment is key to mitigating these quality losses while enhancing drying efficiency.

**Methods:**

The study systematically compared five pretreatments-hot water blanching (HWB), steam blanching (SB), immersion in color-preserving solution (CP), immersion in alkaline solution for reducing toxicity (RT), and hot water blanching combined with ultrasonic treatment (UT)-in terms of drying characteristics, moisture removal, color, bioactive components, safety, microstructure, and flavor.

**Results:**

UT and CP achieved the shortest drying time (5 h), vs. 6 h for HWB and SB, and 8h for RT and CK. NMR revealed that UT accelerated the removal of free water, accounting for its fastest drying rate. CP exhibited multifunctional superiority, which preserved bright color, chlorophyll and carotenoids, while also inhibiting browning, reducing 5-hydroxymethylfurfuralformation, and retaining polyphenols. In addition, all five pretreatments can significantly reduce the colchicine content. UT preserved microstructure but caused excessive chlorophyll retention, resulting in an undesirable greenish hue that deviates from the commercial golden-yellow standard.

**Discussion:**

Considering overall quality, safety, and market acceptability, CP is recommended as the optimal pretreatment for industrial HAD of daylily, whereas UT should be applied only with additional de-greening measures. These findings provided a scientific basis for selecting pretreatment strategies that balance drying efficiency with final product quality.

## 1 Introduction

China possesses abundant resources of daylilies (*Hemerocallis citrina*), with major production areas including Qingyang in Gansu, Datong in Shanxi et al. (1). As a traditional medicinal and edible plant, daylilies are abundant in characteristic secondary metabolites including phenolic compounds, flavonoids, carotenoids and ascorbic acid, and alkaloids, which endow it remarkable health-promoting and dietary therapeutic functions (2, 3). However, the harvesting period of daylilies is notably brief, spanning only from June to August annually. And it exhibited high respiratory activity and is susceptible to spoilage (4). These factors posed prominent challenges to maintaining a stable supply and efficient commercial distribution. Mechanical drying can effectively inhibit microbial growth and enzymatic reactions by substantially reducing water activity, extending the shelf life of products (5). Importantly, specific drying processes can remarkably reduce the content of potential toxic substances in daylilies, such as colchicine, enhancing the food safety (6). Currently, the industry predominantly relied on traditional hot air drying (HAD), which caused issues such as browning and dullness in color, loss of volatile flavor compounds, poor rehydration capacity, and degradation of nutritional components (4). These factors ultimately diminish the commercial value and consumer acceptance of dried daylilies.

Blanching is of great significance for enzyme inactivation, improving texture, color protection, enhancing nutritional value and drying efficiency (7). Different pretreatments have a significant impact on the sensory quality and nutritional components of daylilies. Hot water blanching (HWB) is widely used to rapidly inactivate endogenous enzymes such as polyphenol oxidase, effectively inhibiting enzymatic browning (8). However, the flower buds of daylilies was easily to scatter due to thermal and hydraulic action during blanching, which affected the drying effect and decreased the commercial value. Therefore, HWB was not suitable for the industrial processing of daylilies. In contrast, steam blanching (SB) was more commonly method in the industry. It underwent heat treatment through saturated steam, achieving a similar enzyme inactivation effect while reducing the loss of water-soluble nutrients and flavor compounds, better maintaining the original form and quality of the material (9). Fang et al. (10) reported that SB for 30 s effectively inhibited browning and minimized the loss of phenolic compounds and amino acids in apple slices. However, heat-dominated pretreatments such as HWB or SB inevitably led to the degradation of heat-sensitive nutrients, loss of volatile flavor compounds, and partial pigment deterioration in daylilies (11). In recent years, non-thermal technologies like ultrasound-assisted treatment have demonstrated unique potential. Xu et al. (12) used ultrasound assisted blanching to daylilies and found that the contents of chlorophyll, carotenoids, phenolic compounds, and ascorbic acid in the dried product were higher than that in the hot water blanched sample. Ultrasound improved the overall quality of the dried sample by promoting texture softening, moisture diffusion and microchannels formation (13).The cavitation effect generated by ultrasound not only enhanced mass transfer and improved drying efficiency but enabled the modulation of cellular structure under mild processing conditions (14). Although studies have shown that ultrasound is superior to traditional blanching in retaining pigments such as carotenoids and chlorophyll, an excessively high retention rate of chlorophyll can impart a greenish hue to dried daylilies (15, 16). This deviated from the bright golden color typically preferred in the market. Chemical dipping was widely utilized to decompose the natural wax layer on the surface of materials, which can significantly improve drying efficiency and reduce the degradation of color quality (17). An et al. (18) treated blueberries with ethyl oleate for 5 min, compared with the control, the drying time was reduced by 40.70%, while the total phenolic compounds, total anthocyanin content, and antioxidant activity of the dried samples increased by 37.74%, 47.83%, and 30.75%, respectively. Although chemical dipping pretreatment demonstrated potential in enhancing the drying efficiency and quality of fruits, the potential risk of chemical residues necessitated strict monitoring. Therefore, exploring suitable pre-treatment techniques for daylilies had a prominent practical importance for balancing processing efficiency, safety, and overall quality, as well as for promoting the high-value utilization of this distinctive resource.

The study aimed to systematically investigate the effects of various pretreatments (including HWB, SB, immersion in a combined color-protecting solution, immersion in an alkaline solution, and ultrasound-assisted treatment) on the drying characteristics, moisture removals, color, content of pigments and browning substrates, flavor and microstructure of daylilies. The study sought to identify pretreatment strategies capable of synergistically optimizing processing efficiency and product quality, providing a theoretical foundation for the development of high-quality drying technologies for daylilies.

## 2 Materials and methods

### 2.1 Materials

The daylilies were purchased from the local market (Juwanjia Supermarket—Jinshui District, Zhengzhou, China) and harvested at commercial ripening in the integrated fields in Huaiyang (Henan, China). Daylilies were selected with uniform size, color, and weight, free from visible blemishes, disease and physical damage.

### 2.2 Pretreatment and drying experiments

Daylilies were subjected to five distinct pretreatments, including hot water blanching (HWB), steam blanching (SB), compound color-preserving immersion (CP), alkaline immersion for reducing toxicity (RT) and hot water blanching combined with ultrasonic treatment (UT), were applied to daylily samples. An untreated sample group (CK) was set as the blank control, generating six experimental groups in total. The initial moisture contents of samples were 92.05 ± 0.26%. After pretreatment, samples were immediately cooled in ice-water to terminate enzymatic activity. Subsequently, all samples [(100 ± 2) g per plate] were subjected to the identical stepwise hot-air drying procedure: dried at 60°C for 2 h, then continuously dried at 50°C until the wet-basis moisture content was reduced to ≤ 10%. To accurately record moisture changes throughout drying, samples were weighed every 0.5 h within the initial 2 h of fast drying, the weighing interval was prolonged to 1 h during the middle slow-diffusion stage, and the frequency was adjusted back to 0.5 h when approaching the target residual moisture to precisely determine the constant-weight drying endpoint. All groups were performed in triplicate. The experimental conditions for each group were detailed in Table 1.

**Table 1:** Experimental conditions for different pretreatments of daylilies.

Group	Pretreatment conditions	Drying conditions
HWB	Water at 100 °C for 2 min (55)	HAD at 60 °C for 2 h; then at 50 °C until moisture content (wet basis) ≤ 10%.
SB	Steam at + 100 °C for 2 min (55)	
CP	Immersion in a compound color-preserving solution (0.5% citric acid + 0.3% ascorbic acid + 0.1% NaHSO3) at 25 °C for 30 min (56)	
RT	Immersion in 1%NaHCO_3_ solution at 25 °C for 30 min (55)	
UT	Sequential hot water blanching (100 °C, 60 s) followed by separate ultrasonic immersion (150 W, 20 min) in 0.1% CaCl_2_ aqueous solution (57)	
CK	No pretreatment	

In UT pretreatment, 0.1% calcium chloride solution was used as the ultrasonic medium; calcium ions cross-link with the pectin in the cell wall of the daylilies, enhancing the tissue structure, preventing damage caused by cavitation, and inhibiting the loss of intracellular pigments and polyphenols.

### 2.3 Drying characteristics

#### 2.3.1 Moisture ratio (MR)

As reported by Diamante and Munro (19), the MR of daylilies was calculated via Equation 1:


$$\begin{array}{l} MR_{\left(t\right)}=\frac{M_{t}-M_{e}}{M_{0}-M_{e}} \end{array}$$ (1)

Where, *M*_0_ is the initial dry-basis moisture content (g/g); *M*_*e*_ is the equilibrium dry-basis moisture content (g/g); *M*_*t*_ is the dry-basis moisture content at drying time *t* (g/g).

As described by Hall (20), the dry-basis moisture content *M* was determined using Equation 2:


$$\begin{array}{l} M=\frac{W_{1}-W_{2}}{W_{2}} \end{array}$$ (2)

Where, *W*_1_ represents the total mass at drying time *t* (g), and *W*_2_ is the mass of dry solids (g).

#### 2.3.2 Drying rate (DR)

The DR of daylilies was calculated using Equation 3 (21):


$$\begin{array}{l} DR=\frac{M_{t1}-M_{t2}}{t_{1}-t_{2}} \end{array}$$ (3)

Where, *M*__*t*_1_ is the dry base moisture content at time *t*_1_ (g/g); *M*__*t*_2_ is the dry base moisture content at *t*_2_ (g/g).

#### 2.3.3 Effective moisture diffusivity (Deff)

The Deff of daylilies was determined using Equation 4 (22):


$$\begin{array}{l} \ln\left[MR\right]=\ln\frac{8}{\pi^{2}}-\frac{\pi^{2}\times D_{eff}\times t}{L^{2}} \end{array}$$ (4)

Where, Deff is the effective moisture diffusivity during drying (m^2^/s), *L* is the thickness of the daylilies (m), and *t* is the drying time (s).

### 2.4 Moisture removal

#### 2.4.1 Transverse relaxation time (T_2_) determination

The moisture removal was analyzed using Nuclear Magnetic Resonance (NMR) Imaging Analyzer (NMI20-040 V-I, Suzhou Neway Analytical Instrument Co., Ltd., Jiangsu, China) following the method described by Wang et al. (23). Blanched daylilies segments of 5 cm length (2.2 g ± 0.5 g) were prepared for analysis during drying at 0, 1, 2, and, 4 h. After calibrating the initial system parameters, samples were placed into specimen tubes. The T_2_ was measured using a multi-echo pulse sequence (Carr–Purcell–Meiboom–Gill, CPMG). The specific instrument parameters were set as follows: echo time 0.2 ms, number of echoes 14,000, waiting time 2,000 ms, scan repetitions 8, sampling frequency 250 kHz.

#### 2.4.2 Magnetic resonance imaging (MRI) detection

Samples dried at 0, 1, 2, and, 4 h were positioned at the center of the radiofrequency coil to acquire hydrogen proton density images. MRI experiments were conducted using dedicated imaging software to obtain structural images (24).

### 2.5 Color measurement

Dried samples were ground and passed through an 80-mesh sieve to ensure accuracy and uniformity in color measurement. The color of the dried samples was evaluated using a colorimeter, with results expressed in terms of lightness (L^*^), red-green value (a^*^), and yellow-blue value (b^*^).

### 2.6 Browning degree

Browning degree was determined according to the method described by Udomkun et al. (25). Briefly, 0.2 g of daylily powder (passed through a 80-mesh sieve) was dissolved in 8 mL of a 2% (v/v) acetic acid solution for 2 h. The mixture was homogenized for 1 min, followed by centrifugation at 10,000 r/min for 10 min. The supernatant was collected, and the absorbance at 420 nm was measured using a UV–visible spectrophotometer (UV-1900, Shanghai Jinghua Technology Instrument Co., Ltd., China). The browning degree was expressed as absorbance units per gram of sample (AU/g).

### 2.7 Chlorophyll and carotenoids content

Pigment content was determined according to the method described by Mounir et al. (26). Briefly, dried samples were ground and passed through an 80-mesh sieve. 0.5 g of the powder was accurately weighed, mixed with quartz sand, and repeatedly ground using 80% aqueous acetone as the extraction solvent until the residue became colorless. The homogenate was filtered, and the filtrate was diluted to a final volume of 50 mL in a volumetric flask. Using 80% aqueous acetone as a blank, the absorbance of the extract was measured at wavelengths of 663, 646, and 470 nm. The contents of chlorophyll a, chlorophyll b, and total carotenoids were calculated using Equations 5, 6, and7, respectively:


$$\begin{array}{lll} \rho_{\text{chlorophyll }a}\left(\text{mg/g}\right) & = & \frac{\left(12.21A_{663\text{nm}}-2.81A_{646\text{nm}}\right)\times V}{m} \end{array}$$ (5)

$$\begin{array}{lll} \rho_{\text{chlorophyll}b}\left(\text{mg/g}\right) & = & \frac{\left(20.13A_{646\text{nm}}-5.03A_{663\text{nm}}\right)\times V}{m} \end{array}$$ (6)

$$\begin{array}{lll} \rho_{\text{carotenoids}}\left(\text{mg/g}\right) & = & \frac{\begin{array}{c} {}[1000A_{470\text{nm}}-3.27\times \\ (12.21A_{663\text{nm}}-2.81A_{646\text{nm}})-104\\ \times \left(20.13A_{646\text{nm}}-5.03A_{663\text{nm}})\right]\times V \end{array}}{m\times 299\times 1000} \end{array}$$ (7)

Where, *A*_663nm_, *A*_646nm_, and *A*_470nm_ are the absorbance at wavelengths of 663, 646, and 470 nm, *V* is the volume of the extract (mL); *m* is the mass of the sample (g).

### 2.8 5-hydroxymethylfurfural (5-HMF) content

The 5-HMF content was measured according to the method described by Udomkun et al. (25). Briefly, dried daylily powder was extracted with ethanol. The extract was reacted with trichloroacetic acid and thiobarbituric acid at 40°C, and the absorbance was measured at 443 nm. The concentration was determined using a 5-HMF standard curve and expressed as μg/g dry weight.

### 2.9 Determination of total phenols content

Total polyphenol content was measured by the Folin-Ciocalteu method (27). Accurately weighed 1.0 g of dried daylily powder (passed through a 80-mesh sieve) was placed into a 50 mL centrifuge tube, and 25 mL of 80% (v/v) methanol solution was added. The mixture was ultrasonically extracted at 40°C for 30 min, followed by centrifugation at 10,000 rpm for 10 min. The supernatant was collected, diluted 3-fold, and then 1 mL of the diluted solution was taken and made up to 10 mL with 80% methanol.

For the gallic acid standard curve, gallic acid solutions at concentrations of 20, 40, 60, 80, 100, and 120 μg/mL were prepared. An aliquot of 0.8 mL of each standard solution was mixed with 4 mL of 10-fold diluted Folin-Ciocalteu reagent, followed by the addition of 6 mL of 10% (w/v) Na_2_CO3 solution with thorough mixing. The mixture was allowed to react at room temperature in the dark for 2 h. The absorbance was measured at 765 nm using 80% methanol as the blank. The standard curve was constructed by plotting absorbance against gallic acid concentration. For sample determination, 0.8 mL of each extracted solution was taken and treated following the same procedure as described above.

### 2.10 Extraction and quantification of colchicine

The extraction and quantification of colchicine were performed according to the method described by Miao et al. (28). Dried samples were ground into powder, and 15 g of the powder was extracted with methanol-water (1:8, v/v) at 30°C for 24 h. This extraction was repeated five times. The combined extracts were filtered and centrifuged at 10,000 r/min for 15 min. The supernatant was evaporated to dryness at 55°C. The residue was dissolved in 5% acetic acid and extracted with petroleum ether to remove non-alkaloid impurities. The acidic aqueous phase was further extracted with ethyl ether, adjusted to pH 9 with ammonium hydroxide, and finally extracted with chloroform. The chloroform phase was evaporated to dryness, and the residue was weighed. The extract was reconstituted in methanol-water and analyzed using an Agilent 1,260 high-performance liquid chromatography (HPLC) system. The HPLC conditions were as follows: column, PL1512-5501 ChromSphe C18 (250 mm × 4.6 mm, 5 μm); flow rate, 0.5 mL/min; injection volume, 10 μL; mobile phase, methanol-water (50:50, v/v); detection wavelength, 350 nm. Pure colchicine standard (C9754, Sigma-Aldrich, St. Louis, MO, USA) was used for calibration.

### 2.11 Scanning electron microscopy (SEM)

The microstructure of dried daylilies was performed using scanning electron microscope (JSM-IT200, Jeol, Japan). The flower bud tissue of the sample were broken naturally and fixed the cross-section upwards on the double-sided conductive tape. After sputtering for 90 s with a layer of gold (10 nm), the SEM images of the cross-section surface of the dried samples were observed at an accelerating voltage of 10 kV under high vacuum condition (10 Pa) and recorded at a magnification of 100 × and 500 × .

### 2.12 Gas chromatography–ion mobility spectrometry (GC-IMS)

Analysis of volatile organic compounds (VOCs) of daylily powder was performed using GC-IMS flavor analyzer (FlavourSpec^®^, G.A.S. GmbH, Germany), following the method described by Xie et al. (29) with minor modifications. The instrument was equipped with an FS-SE-54-CB-0.5 capillary column (15 m × 0.53 mm × 0.5 μm). 1.0 g sample (dry basis) was weighed into a 20 mL headspace vial and incubated at 60°C with agitation at 250 r/min for 20 min. Subsequently, 500 μL of headspace gas was injected using a syringe preheated to 85°C. High-purity nitrogen (99.999%) was used as the carrier gas with the following programmed flow: initial flow of 2 mL/min held for 2 min, increased to 100 mL/min over 20 min, and then maintained for 10 min before termination. The analytes were separated on a column maintained at 60°C and subsequently ionized in the IMS ionization chamber at 45°C. The drift gas flow was kept constant at 150 mL/min. Compound identification was conducted by comparing both the retention indices and drift times of the samples with those of reference standards in the GC-IMS library, supplemented by searching the built-in NIST and IMS databases through the GC × IMS Library Search software. The characteristic fingerprint plots of volatile organic compounds (VOCs) were automatically produced by the Gallery Plot plug-in of VOCal software, which is the built-in data analysis platform matched to the GC-IMS flavor analyzer.

### 2.13 Statistical data analyses

All the data are presented as mean ± standard deviation (*n* = 3). Statistically significant differences (*P* < 0.05) were analyzed by one-way analysis of variance, followed by the Duncan test. The statistical analyses were performed using the SPSS software (version 26.0, SPSS Inc., Chicago, IL, USA). All graphs were drawn using the Origin 2021 software.

## 3 Results and discussion

### 3.1 Effects of pretreatments on drying characteristics of daylilies

**Figure 1:**
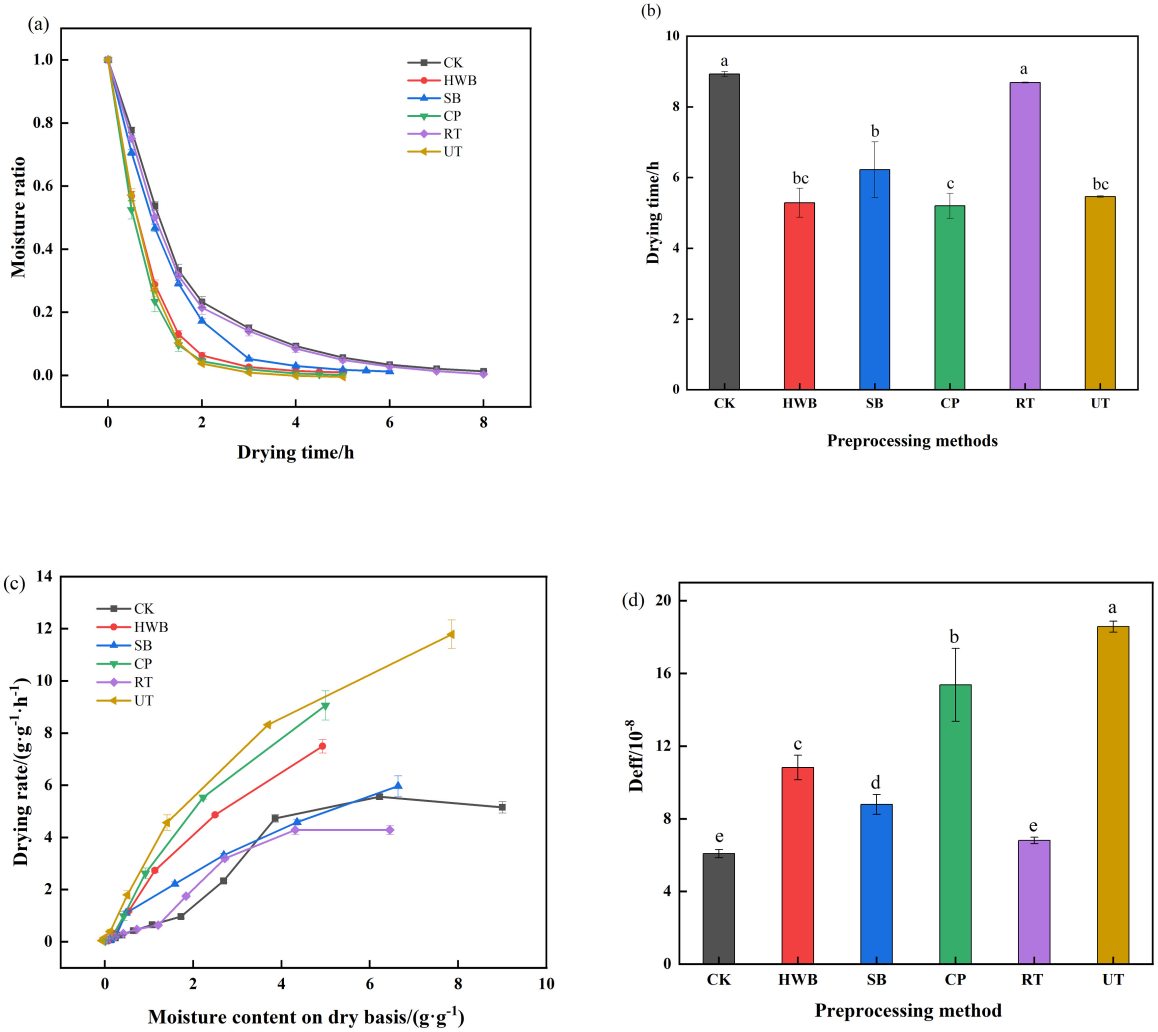
The drying characteristic curves of daylilies during drying in different pretreatments. **(a)**–**(d)** represented the drying time-MR curve, drying time, moisture content (dry basis)-DR curve, and Deff, respectively. The different lowercase letter indicated statistically significant difference (*P* < 0.05), the same applies below.

Figure 1 demonstrated that different pretreatments significantly affected the MR and DR of daylilies (*P* < 0.05). The MR decreased continuously with drying time, while the DR exhibited a decelerating trend. This was because the DR depended on both surface moisture evaporation and internal moisture diffusion. The required drying times for the UT, CP, HWB, SB, RT, and CK were 5, 5, 6, 6, 8, and 8 h, respectively. This variation was attributed to differing degrees of cellular structure disruption and altered moisture removal pathways caused by the pretreatments. In other words, pretreatments can regulate cellular structure to influence DR (17). The UT showed the fastest DR, primarily due to the synergistic effect of blanching at 100°C combined with ultrasound, which created microscopic moisture channels (30). The CP ranked second, as the citric-ascorbic acid system preserved color and enhanced cell membrane permeability, promoting moisture diffusion (31). The RT was only slightly faster than CK, as its main function was degrading colchicine under alkaline conditions with minimal effect on permeability. The CK was slowest, relying solely on slow molecular diffusion for moisture removal. In summary, pretreatments reduce water-binding capacity and establish effective moisture removal channels, making them key to regulating DR. UT and CP demonstrated optimal performance in improving drying efficiency.

### 3.2 Effects of pretreatments on moisture removal of daylilies

**Figure 2:**
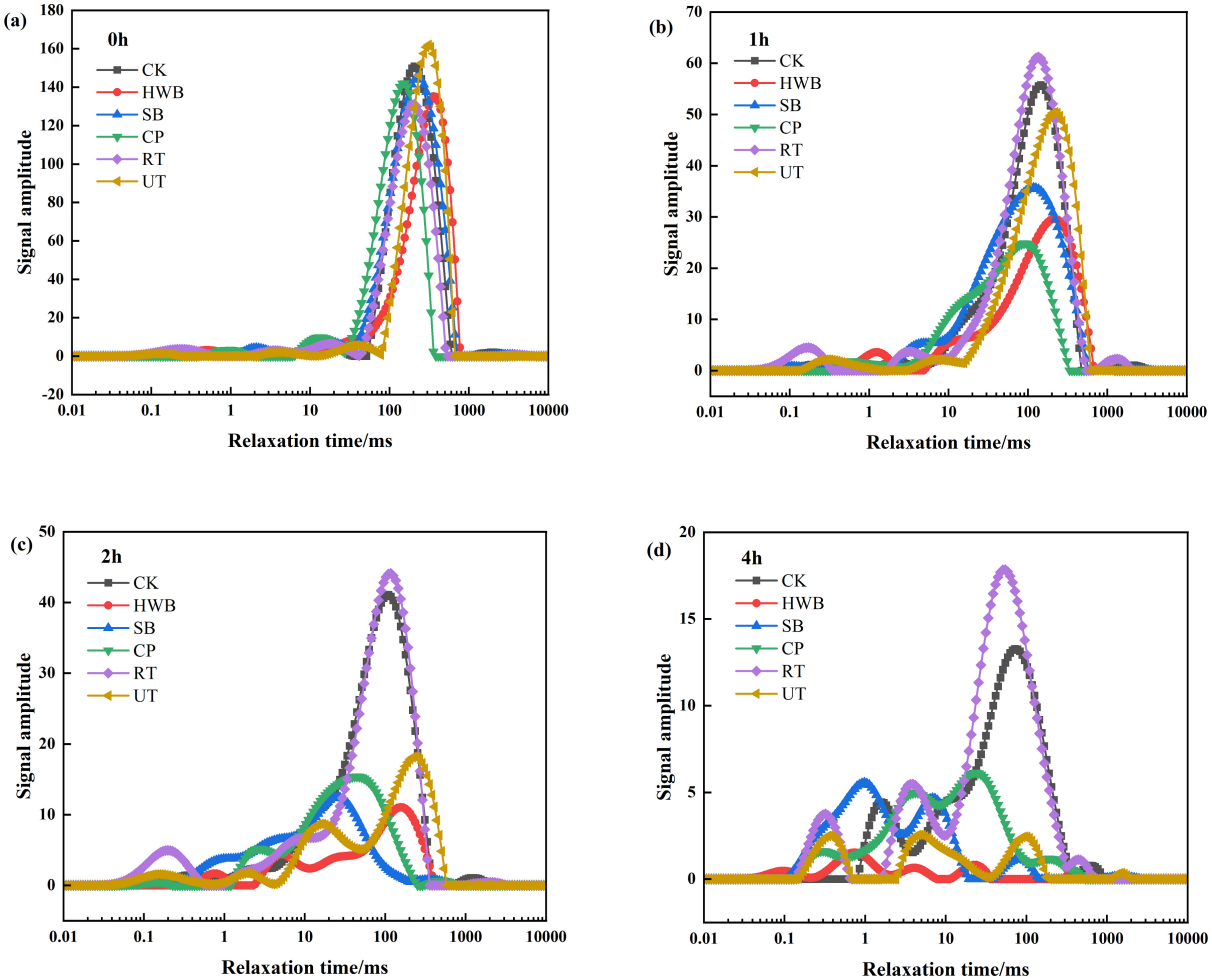
Inversion of T_2_ during drying of daylilies in different pretreatments. **(a)**–**(d)** represented the T_2_ inversion spectra dried at 0, 1, 2, and 4 h, respectively. Each subfigure was independently normalized for peak position analysis.

Low-field NMR can rapidly, directly, and non-destructively determine water distribution in foods and the mobility of moisture protons associated with various molecules (32). Figure 2 showed the changes in T_2_ relaxation times of water in daylilies under different pretreatments. The LF-NMR spectra exhibited three distinct transverse relaxation peaks T_21_, T_22_, and T_23_, which correspond to bound water, semi-bound water, and free water within cell tissues, respectively. As shown in Figure 2a, at 0 h of drying, the moisture states varied among pretreatments, indicating distinct initial effects on cellular structure and water-binding states. Different pretreatments can influence water-binding properties and drying characteristics by altering cell membrane permeability (33). During drying, the T_2_ spectra of all groups shifted leftward with decreasing signal intensity, reflecting continuous moisture loss, particularly free water. After 1 h of drying (Figure 2b), the T_2_3 peak of the UT decreased most noticeably, indicating the fastest free water removal, attributed to the enhanced water mobility from ultrasound and blanching. The RT and CK showed slower DR. By 2 h (Figure 2c), the T_2_3 peak of the UT nearly disappeared, with signals concentrated in the T_2_1 and T_22_ regions, suggesting most free water had been removed due to improved cell permeability and efficient moisture channels. In contrast, the CK retained a clear T_2_3 peak with more residual free water. After 4 h (Figure 2d), the moisture signal of the UT was mainly in the bound and semi-bound water regions, indicating substantial removal of internal free water, further confirming the promoting effect of ultrasound pretreatment on moisture removal. The CK still showed a distinct T_2_3 signal, reflecting the slowest dehydration progress due to reliance on slow diffusion (34). In summary, UT significantly accelerated free water removal, explaining the fastest DR from a moisture-state perspective.

**Figure 3:**
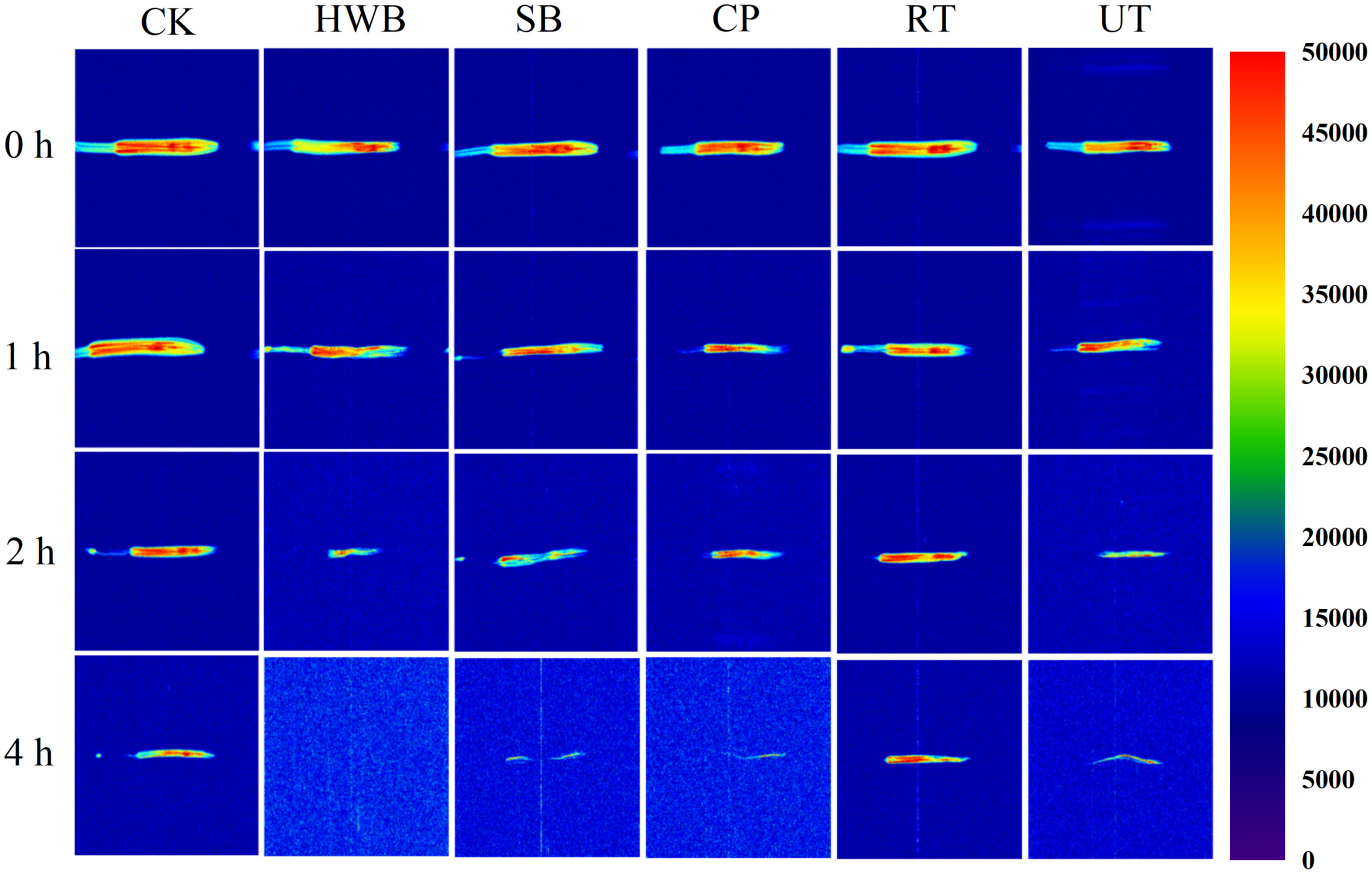
MRI images of daylilies during drying process in different pretreatments. Scale bar indicates relative signal intensity (a.u., arbitrary units), which is directly proportional to the proton density (i.e., the relative water content) within each voxel. These values are used for relative comparison among different regions within the same image and across samples measured under identical parameters.

As shown in Figure 3, MRI was used to dynamically visualize water distribution in daylilies under different pretreatments during drying. At the initial stage (0 h), all groups showed strong red/yellow signals, indicating high and uniform moisture, while only the UT exhibited slightly lighter signals, suggesting reduced water binding after ultrasonic (35). At 1 h of drying, signal intensity generally decreased across all groups, but differences began to emerge. The red/yellow areas in the UT contracted rapidly, indicating significant moisture removal, while the CK and RT retained more concentrated high-signal zones, suggesting slower moisture removal. By 2 h of drying, the moisture distribution in the UT had narrowed noticeably. The HWB retained only faint yellow-green signals, whereas the CK and RT maintained clear red/yellow areas, with significantly higher residual moisture compared to other groups. After 4 h of drying, the HWB appeared dark blue, indicating that free water was largely removed. The CP, SB, and UT showed extremely weak signals. In contrast, the RT still displayed noticeable red/yellow signals, representing the highest residual moisture and slowest dehydration rate among all treatments. The CK showed faint light-yellow signals, with a dehydration progress faster than that of the RT. The RT primarily focused on chemical detoxification, had a weaker effect on improving cell permeability and moisture channels. Consequently, moisture removal was hindered, leading to the highest residual moisture and the lowest DR (36). This conclusion aligns with and mutually corroborates the attenuation pattern of the T_2_3 peak observed in the NMR analysis, together revealing the internal mechanism by which different pretreatments influence drying efficiency through regulating moisture removal states.

### 3.3 Effects of pretreatments on color of daylilies

Color is a key indicator of the sensory quality and commercial value of dried daylilies. Its final appearance was determined by enzymatic browning, non-enzymatic reactions, and pigment degradation/retention during drying (37). Dried daylilies are predominantly preferred with a bright golden-yellow color, which allow the underlying carotenoids to become visually dominant. Therefore, the carotenoids are the most important color-related pigments, while excessive chlorophyll retention is considered a quality defect rather than a nutritional advantage in terms of sensory evaluation. The effects of different pretreatments on the color of daylilies were shown in Table 2. In terms of brightness (L^*^), the CP exhibited the highest L^*^ value. This was because the combined color-preservation system of citric acid and ascorbic acid used in CP effectively reduced polyphenol oxidase (PPO) and peroxidase (POD), the core enzymes triggering enzymatic browning slowed down the browning process, and maintained the light reflectivity of the cellular structure, resulting in the highest sample brightness. Notably, browning during daylily drying is driven by both enzymatic oxidation and non-enzymatic pathways including Maillard reaction and carotenoid thermal degradation; ascorbic acid additionally inhibits non-enzymatic browning by scavenging reactive carbonyl intermediates (58). The SB, CK, and RT followed, with relatively close L^*^ values, whereas HWB and UT showed the lowest brightness. Regarding the red-green index (a^*^), the UT had the lowest a^*^ value, indicating the most pronounced green characteristics. This was “to the effect of Ca^2+^ cross-linking with pectin in the cell walls during UT, which reduced the dissolution and degradation of chlorophyll (38). From a commercial processing perspective, this excessive chlorophyll retention is highly undesirable, as it results in an incomplete “de-greening” process, giving the dried product a greenish hue that fails to meet the market standard of golden-yellow daylilies. In contrast, the a^*^ values of the CP and HWB were −1.75 and −1.24, indicating a more favorable pigment profile where chlorophyll has been sufficiently degraded and carotenoids are well preserved. In terms of the yellow-blue index (b^*^), the CP and SB showed the highest b^*^ values, presenting the most desirable golden-yellow appearance. This superior yellowness is not solely due to ascorbic acid slowing carotenoid oxidative degradation, but more importantly, because these two pretreatments effectively promoted chlorophyll degradation, allowing the yellow carotenoid color to be fully unveiled (39). Conversely, the RT and UT had the lowest b^*^ values. This was mainly attributed to its excessively high chlorophyll retention, which severely masked the yellow color derived from carotenoids. The results indicated that different pretreatments significantly affected the L^*^, a^*^, and b^*^ values of daylilies. Notably, the key to achieving optimal color lies not simply in maximizing total pigment retention, but in striking a balance between chlorophyll degradation and carotenoid preservation to meet the commercial quality requirements.

**Table 2:** Effects of different pretreatments on color parameters and images of daylilies.

Group	L^*^	a^*^	b^*^	Image
CK	69.09 ± 1.48^b^	−0.18 ± 0.37^a^	31.94 ± 1.21^b^	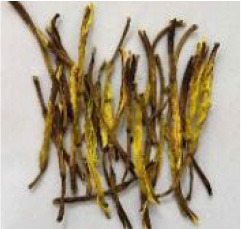
HWB	66.06 ± 0.67^c^	−1.24 ± 0.66^bc^	31.79 ± 0.84^b^	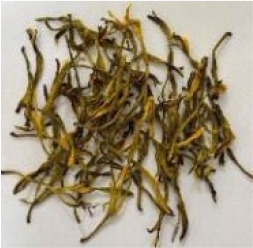
SB	69.73 ± 0.39^b^	−0.49 ± 0.48^ab^	34.84 ± 0.49^a^	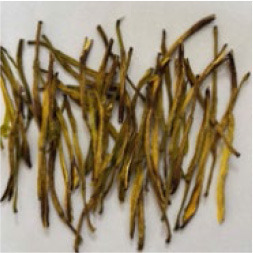
CP	72.96 ± 0.37^a^	−1.75 ± 0.37^c^	34.93 ± 0.38^a^	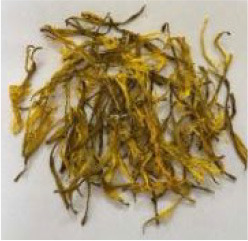
RT	68.56 ± 1.60^b^	−0.51 ± 0.19^ab^	29.50 ± 1.51^c^	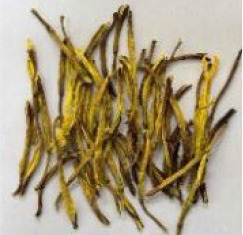
UT	64.28 ± 1.48^c^	−4.46 ± 0.50^d^	29.15 ± 1.05^c^	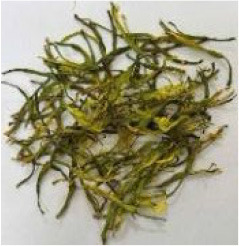

Different lowercase letters represent significant differences between groups (same column, P < 0.05). The “*” symbol conforms to the specification parameters defined by CIE 1976, emphasizing its optimization based on human visual uniformity.

### 3.4 Effects of pretreatments on chlorophyll and carotenoids content of daylilies

**Figure 4:**
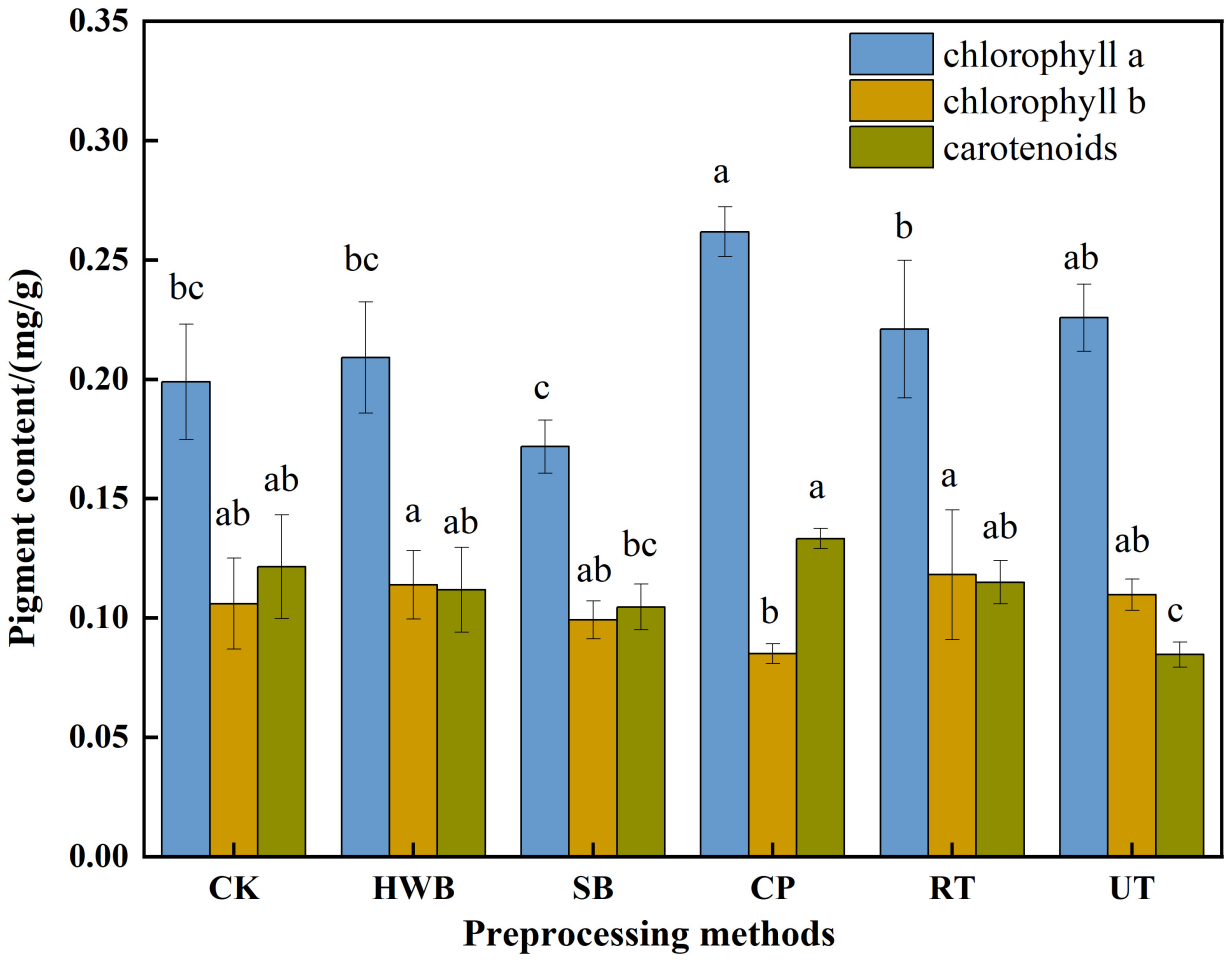
Effects of different pretreatments on pigment content of daylilies.

Figure 4 showed the changes in chlorophyll and carotenoid content in daylilies under different pretreatments. Regarding chlorophyll retention, the CP demonstrated the best overall effect on pigment preservation. The CP showed the highest relative content of chlorophyll a, which increased by 24% compared to the CK. This was mainly attributed to the synergistic effect of citric acid and ascorbic acid in its color-protecting solution, effectively inhibiting chlorophyllase activity and the removal of magnesium ions (40). In terms of carotenoid retention, the CP exhibited a clear advantage, with the highest carotenoid content. After RT, the overall pigment content remained lower than that of the CP, indicating that the alkaline environment, while degrading toxins, also affected pigment stability to some extent. The UT resulted in the lowest carotenoid content, possibly because ultrasonication promoted the dissolution of carotenoids. Zhang et al. (16) reported that compared to untreated daylilies, ultrasonication reduced carotenoid content (lutein, zeaxanthin, β-carotene). In contrast, SB led to significant pigment degradation, with both chlorophyll a and carotenoid contents lower than those in the CK, showing reductions of 13.64% and 13.91%, respectively. During the later stages of heat treatment, the diminished color of daylilies can be attributed to the chemical degradation of chlorophylls and the loss of free pigments due to leaching into the blanching water (41). Therefore, the CP best preserved the pigment composition and content in daylilies.

### 3.5 Effects of pretreatments on browning degree, total phenols content and 5-HMF of daylilies

**Figure 5:**
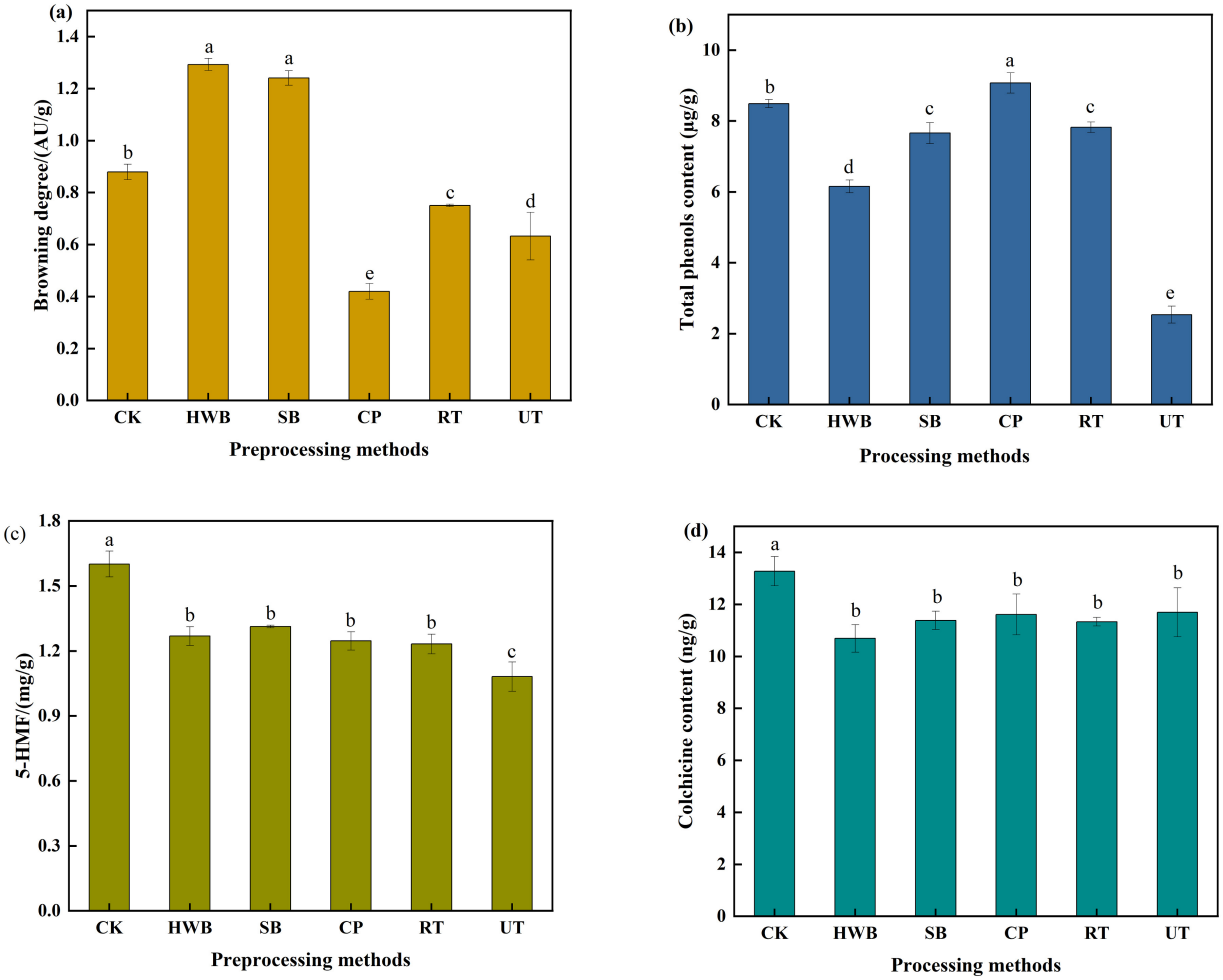
Effects of different pretreatments on browning degree **(a)**, total phenols content **(b)**, 5-HMF content **(c)** and colchicine content **(d)** of daylilies.

The browning index of daylilies subjected to different pretreatments was shown in Figure 5a. The pretreatments significantly affected the browning coefficient of daylilies (*P* < 0.05). In terms of browning inhibition, daylilies treated with CP exhibited the lowest browning index, indicating optimal suppression of browning. In contrast, the two thermal treatments (HWB and SB) induced the most severe browning, with browning indices significantly higher than that of CK. This is likely because the relatively high temperatures during pretreatment promoted enzymatic browning, leading to increased formation of browning products (42). The total phenols content in daylilies under different pretreatments was shown in Figure 5b. Regarding the retention of total phenols, the CP demonstrated a significant advantage, yielding the highest total phenols content. The added citric acid and ascorbic acid acted as antioxidants, directly protecting phenolic compounds from oxidation and inhibiting relevant enzyme activity by lowering the pH (43). The total phenols content in the two conventional thermal pretreatments (HWB and SB) was significantly lower than that in the CP. Although high temperatures can rapidly inactivate enzymes, they may also cause thermal degradation or leaching losses of some heat-sensitive phenolic compounds. Daylilies subjected to UT had the lowest total phenols content, even lower than the CK, possibly because the ultrasonic pretreatment disrupted the tissue structure of daylilies, leading to the leaching of cellular contents. The 5-HMF content in daylilies under different pretreatments was shown in Figure 5c. All five pretreatments effectively reduced the 5-HMF content, indicating that the pretreatment conditions could all inhibit 5-HMF formation to some extent. The UT performed best, resulting in the lowest 5-HMF content, which was significantly superior to conventional blanching processes. Consistent with the findings of Zhang et al. (16), the 5-HMF content in ultrasonically pretreated daylilies was lower than that in heat-treated samples. Therefore, the CP can simultaneously and effectively inhibit browning, reduce the formation of 5-HMF, and better preserve phenolic compounds.

### 3.6 Effects of pretreatments on colchicine content of daylilies

Colchicine is a toxic alkaloid that was originally isolated and determined from plants of colchicum. Parekh et al. (44) reported that the maximum safe daily intake of colchicine for adults is 2.4 mg, and any consumption exceeding this threshold is considered overdose. Meanwhile, acute ingestion exceeding 0.5–0.8 mg/kg body weight carries fatal risks. The colchicine content in daylilies subjected to different pretreatments was shown in Figure 5d. All five pretreatments effectively reduced the colchicine content in the dried daylilies, thereby enhancing its safety for consumption. The CK exhibited the highest colchicine content, whereas the colchicine levels in the other five pretreatments were significantly reduced to comparable levels. These results indicated that the five pretreatments were similarly effective in lowering colchicine content, with no statistically significant differences observed among the five pretreatments (*P* > 0.05).

### 3.7 Effects of pretreatments on microstructure of daylilies

**Figure 6:**
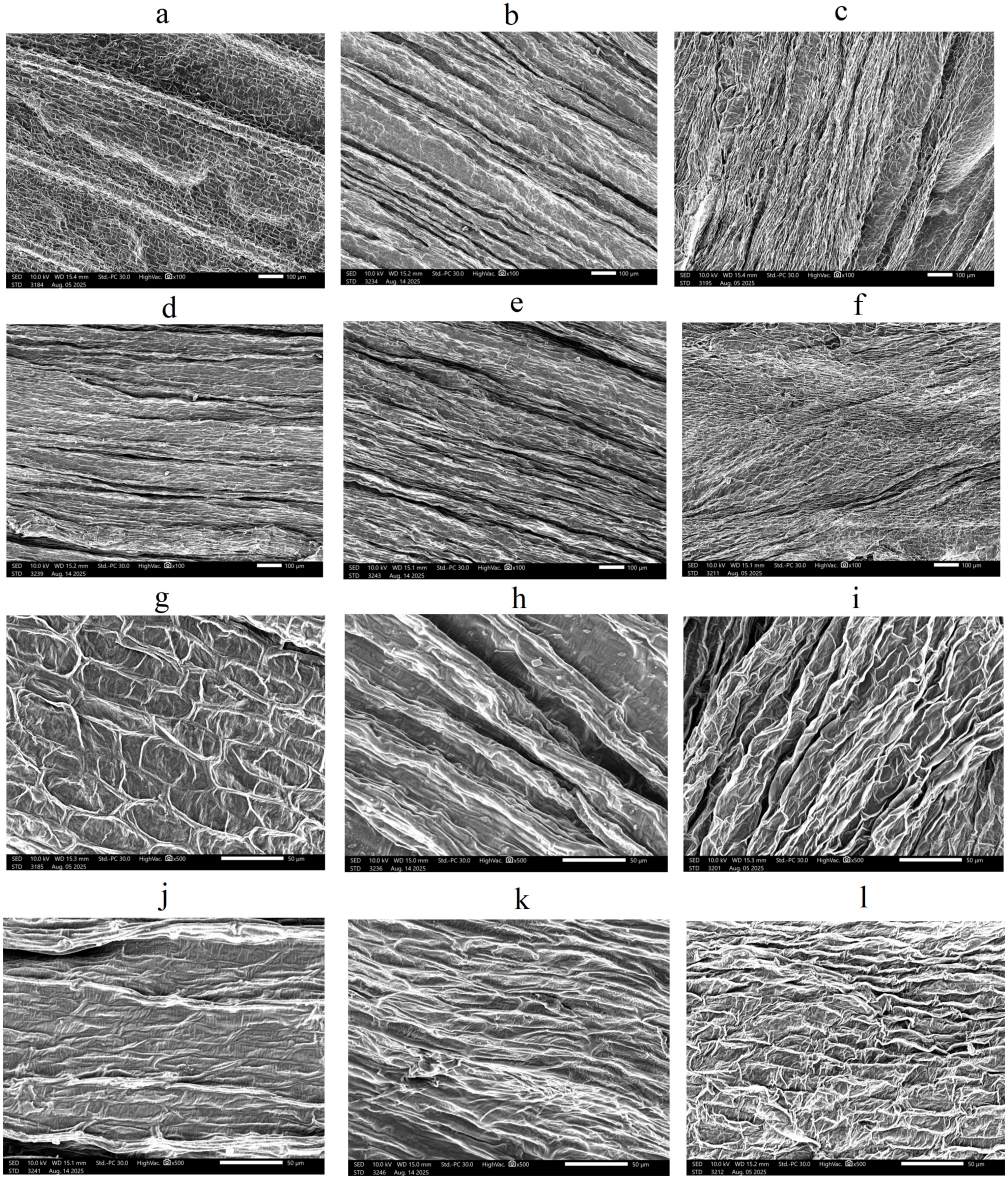
SEM of daylilies in different pretreatments. All samples were taken from the natural fracture cross-section of flower buds. **(a)**–**(f)** were the SEM images of daylilies processed with CK, HWB, SB, CP, RT and UT at 100× magnification, respectively. **(g)–(l)** were the SEM images of daylilies processed with CK, HWB, SB, CP, RT and UT at 500× magnification, respectively. Scale bars are indicated on each image.

Figure 6 showed that different pretreatments have a significant impact on the changes in the microstructure of daylilies. Compared with the CK, the HWB and RT suffered the most severe surface structural damage, as corroborated by SEM. For the HWB, damage was resulted from heat-induced rapid tissue cell water loss, protoplast denaturation, coagulative shrinkage and cell wrinkling, disrupting the original cellular orderly arrangement. The RT showed more drastic structural destruction: besides the alkaline environment damaging the permeability of cell membranes, the alkali solution further hydrolyzes key components of cell walls such as cellulose and pectin, undermining the structural scaffolding of tissue cells (45). The SB had milder cell shrinkage than HWB and RT, with blurred outlines and local debris; moist heat induced thermal damage, impairing microstructural integrity (4). The CP exhibited a uniform texture similar to CK, with only mild cell wall folding, likely due to the synergistic protection afforded by gentle processing and stabilizers. The UT had slightly rough surfaces with subtle micropores but intact cellular integrity; ultrasonic cavitation and vibration caused only localized physical effects without extensive structural damage (46). These microstructural discrepancies correlated directly with the retention of bioactive compounds. Combined with previous results, the CP exhibited the highest total phenols and carotenoids, which aligned with its well-preserved cellular structure that minimized the leakage of intracellular bioactive substances. Notably, the UT had the lowest total phenol content among all treatments, likely due to the localized physical effects of ultrasound causing a loss of phenolic compounds. In contrast, samples with severe structural damage (e.g., RT and SB) underwent pronounced texture deterioration and lower retention of bioactive compounds. Collectively, these findings demonstrated that pretreatments maintaining cellular integrity (e.g., CP and UT) better preserve bioactive components and textural properties.

### 3.8 Effects of pretreatments on flavor of daylilies

**Figure 7:**
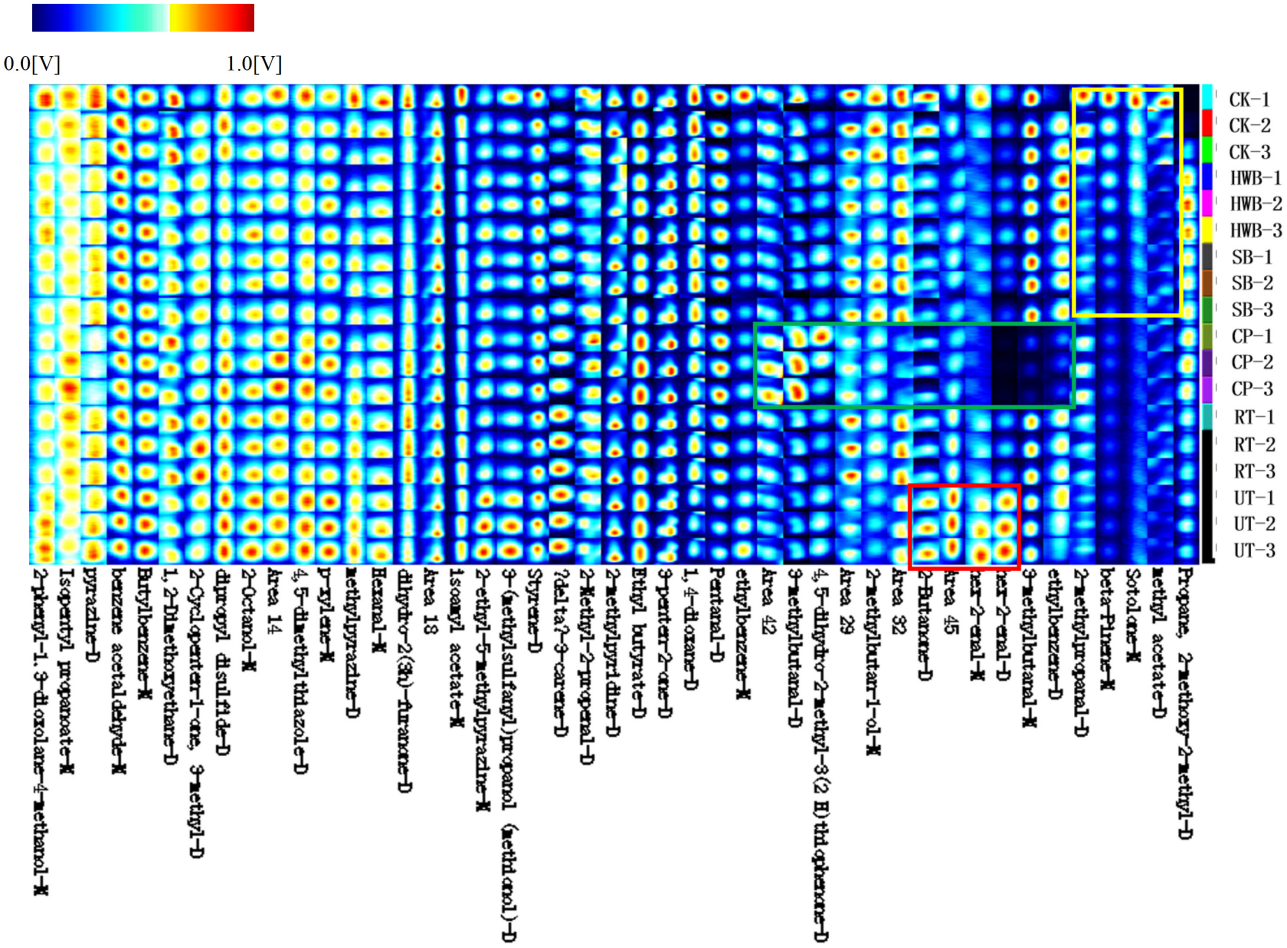
Fingerprinting spectrogram of VOCs of daylilies in different pretreatments. The scale represents the strength of the current signal, and the original data unit is volts (V). The built-in software of GC-IMS converts the original detector current into a chromatogram after pseudo color processing based on signal strength (peak volume). Each row corresponds to a pretreatment group, and each column corresponds to a compound listed in the Table 3. The color intensity indicates the relative normalized signal intensity (peak volume %) of each compound, enabling direct semi-quantitative comparison among treatments.

The results of the VOCs of the dried daylilies under different pretreatments were shown in Table 3. Table 3 provided the identification parameters (e.g., retention index, retention time, drift time, and odor description) for all compounds detected. The variations in the relative content of these compounds among the 6 groups were displayed in the fingerprinting spectrogram (Figure 7), where the intensity of each spot reflects its abundance in each specific treatment. A total of 45 volatile flavor substances were identified by the built-in NIST database and IMS database in GC × IMS Library Search, and 39 flavor substances (including dimers) were obtained qualitatively, which accounted for 86.68% of the total. The variations in VOCs among different pretreated dried daylilies can be attributed to the distinct impacts of each pretreatment on key biochemical pathways governing flavor formation during subsequent drying (27). In the present study, 5 aromatic hydrocarbons, 4 alcohols, 6 ketones, 4 esters, 9 aldehydes and 11 others were detected. The predominance of aldehydes primarily stemmed from lipid oxidation (via autoxidation or enzymatic pathways initiated during tissue disruption) and the Strecker degradation of amino acids, often accelerated by thermal pretreatment (47). For instance, HWB and SB, while effectively deactivating enzymes, likely promote thermal oxidation of unsaturated fatty acids (48), leading to elevated levels of pentanal, hexanal, benzene acetaldehyde and hex-2-enal (presenting grassy, fresh and sweet fruity). The presence of pyrazines was a classic indicator of the Maillard reaction and associated Strecker degradation, facilitated by the heat and moisture conditions during drying (49). Pretreatments like SB and UT provide an ideal environment for these reactions by increasing reactant availability through cell wall disruption or moderate heating, yielding nutty, roasted notes. Alcohols and ketones often arisen as secondary products of lipid oxidation or via microbial and enzymatic pathways, while esters typically form through esterification reactions between acids and alcohols, potentially influenced by tissue disruption from ultrasonication (50, 51). Importantly, chemical pretreatments such as RT or CP can alter the pH or introduce antioxidants, thereby modulating the Maillard reaction and oxidation rates while preserving more desirable volatiles. Different pretreatments significantly affected the generation pathways and relative contents of various volatiles, which led to the flavor differences of the final dried products.

**Table 3:** Identification parameters of VOCs detected in daylily samples across all pretreatment groups.

Count	Compound	CAS#	Formula	MW	RI	Rt [sec]	Dt [a.u.]	Comment
Aromatic hydrocarbons								
1	styrene	C100425	C_8_H_8_	104.2	891.8	482.333	1.41581	special aromatic aroma, organic, sweet, pungent
2	ethylbenzene-M	C100414	C_8_H_10_	106.2	865.5	429.146	1.07925	Aromatic odor
3	ethylbenzene-D	C100414	C_8_H_10_	106.2	850.8	402.092	1.4519	Aromatic odor
4	butylbenzene	C104518	C_10_H_14_	134.2	1,048.6	814.313	1.21021	sweet, bitter almonds
5	p-xylene	C106423	C_8_H_10_	106.2	866.2	430.545	1.05854	aromatic
Alcohols								
6	2-octanol	C123966	C_8_H_18_O	130.2	971.9	654.834	1.44261	fresh, herbaceous, earthy
7	2-methylbutan-1-ol	C137326	C_5_H_12_O	88.1	714.9	216.996	1.23321	roast onion, fruity, floral, wine
8	2-phenyl-1.3-dioxolane-4-methanol	C1708390	C_10_H_12_O_3_	180.2	972.1	655.49	1.14914	sweet berries, bitter almonds
9	3-(methylsulfanyl)propanol	C505102	C_4_H_10_OS	106.2	971.9	654.834	1.46784	strong scallions, meat, soy sauce
Ketones								
10	2-butanone	C78933	C_4_H_8_O	72.1	573.8	135.781	1.24552	fruity, camphor
11	3-penten-2-one	C625332	C_5_H_8_O	84.1	741.9	245.959	1.34327	Fruity, turns into spicy during storage
12	dihydro-2(3h)-furanone	C96480	C_4_H_6_O_2_	86.1	898.5	494.93	1.30707	Cream, fat, caramel
13	4,5-dihydro-2-methyl-3(2H)thiophenone	C13679851	C_5_H_8_OS	116.2	970.1	650.423	1.54359	
14	sotolone	C28664359	C_6_H_8_O_3_	128.1	1,105.7	932.98	1.21835	sweet, caramel, maple
15	2-cyclopenten-1-one, 3-methyl	C2758181	C_6_H_8_O	96.1	971.9	654.834	1.41605	Fruity
Esters								
16	methyl acetate	C79209	C_3_H_6_O_2_	74.1	539.1	122.25	1.19213	ethereal
17	ethyl butyrate	C105544	C_6_H_12_O_2_	116.2	792	309.94	1.55925	pineapple, fruity, ester, whiskey
18	isoamyl acetate	C123922	C_7_H_14_O_2_	130.2	880	457.839	1.30604	sweet, banana, fruity
19	isopentyl propanoate	C105680	C_8_H_16_O_2_	144.2	972.7	656.802	1.34435	fruity
Aldehydes								
20	pentanal	C110623	C_5_H_10_O	86.1	698.5	201.179	1.42277	green grassy, faint banana, pungent
21	hexanal	C66251	C_6_H_12_O	100.2	798	318.266	1.26528	fresh, green, fat, fruity
22	benzene acetaldehyde	C122781	C_8_H_8_O	120.2	1,048.3	813.845	1.25175	hyacinth, sweet fruity, almond, cherry, clover honey, cocoa
23	hex-2-enal-M	C505577	C_6_H_10_O	98.1	853.7	407.373	1.17933	sweet almonds, fruity, green leaves, apples, plums, vegetables
24	hex-2-enal-D	C505577	C_6_H_10_O	98.1	852.4	405.037	1.51277	sweet almonds, fruity, green leaves, apples, plums, vegetables
25	3-methylbutanal-M	C590863	C_5_H_10_O	86.1	645.7	168.734	1.19827	Chocolate, fat
26	3-methylbutanal-D	C590863	C_5_H_10_O	86.1	635.8	163.75	1.40687	Chocolate, fat
27	2-methylpropanal	C78842	C_4_H_8_O	72.1	553.7	127.768	1.28122	banana, melon, slightly nutty
28	2-methyl-2-propenal	C78853	C_4_H_6_O	70.1	566.4	132.753	1.21954	hyacinth foliage
Others								
29	1,4-dioxane	C123911	C_4_H_8_O_2_	88.1	719.7	221.898	1.32949	pungent, ether
30	1,2-dimethoxyethane	C110714	C_4_H_10_O_2_	90.1	642.5	167.098	1.31202	ether
31	propane, 2-methoxy-2-methyl	C1634044	C_5_H_12_O	88.1	537.8	121.765	1.3609	fruity, ether
32	β-pinene	C127913	C_10_H_16_	136.2	979.1	673.198	1.21687	resin, green
33	δ-3-carene	C13466789	C_10_H_16_	136.2	1,003.8	732.052	1.67469	Citrus, lemon, woody
34	pyrazine	C290379	C_4_H_4_N_2_	80.1	716.8	218.955	1.27376	nut, musty
35	methyl pyrazine	C109080	C_5_H_6_N_2_	94.1	817.6	347.134	1.09778	nutty, moldy, roast, earthy
36	2-ethyl-5-methylpyrazine	C13360640	C_7_H_10_N_2_	122.2	993.6	711.374	1.17574	Coffee, nutty, roasted, green grassy
37	2-methylpyridine	C109068	C_6_H_7_N	93.1	811.8	338.264	1.34951	Pyridine
38	4,5-dimethylthiazole	C3581917	C_5_H_7_NS	113.2	929.2	556.515	1.48208	Green, Nut, Roast
39	dipropyl disulfide	C629196	C_6_H_14_S_2_	150.3	1,108.5	939.293	1.4776	sulfury, onion

The table serves as the compound identification library. The relative abundance and treatment differences of these compounds were visualized in the fingerprinting spectrogram (Figure 7). The letters M and D represent the monomers and dimers of a compound, respectively.

The fingerprints of VOCs in daylilies under different pretreatments were shown in Figure 7. The changes of VOCs under different pretreatments were indicated by different colored frames. The high similarity in VOCs observed in the CK, HWB, and SB stemmed from their shared reliance on thermal mechanisms, which rapidly inactivated endogenous enzymes and created substrates for subsequent drying-driven reactions, predominantly the Maillard reaction and generalized lipid oxidation (52). This explained the marked reduction or absence of compounds in all pretreated samples such as propane, 2-methoxy-2-methyl, methyl acetate, and sotolone, which accumulated in the CK through prolonged thermal degradation. The unique profiles emerged from treatments that deviated from conventional thermal processing (in yellow box). Specifically, the UT exhibited significantly elevated levels of 2-butanone and hex-2-enal, a direct consequence of ultrasonic cavitation physically disrupting cellular structures (in red box). This disruption liberated and activated lipoxygenase, thereby intensifying specific pathways of enzymatic lipid peroxidation (53). Conversely, the CP displayed a chemically modulated results, characterized by elevated concentrations of 3-methylbutanal-D and 4, 5-dihydro-2-methyl-3(2H)-thiophenone, coupled with the near absence of compounds like 2-methylbutan-1-ol and hex-2-enal (in green box). This selective enhancement and suppression were driven by the antioxidant and pH-regulating components in the soaking solution, which redirected the Maillard/Strecker degradation pathways and inhibited specific branches of lipid oxidation (54). In summary, the VOCs profile precisely reflected the underlying pretreatment mechanisms, wherein thermal methods homogenized the reaction environment, physical treatment (UT) amplified enzymatic oxidation, whereas chemical pretreatment (CP) selectively channeled and suppressed core reactions, thereby shaping the final flavor profile.

## Conclusions

The study comprehensively analyzed the effects of five pretreatments (HWB, SB, CP, RT, UT) on the drying characteristics, moisture removal, color, pigment content, browning products, microstructure, and flavor profile of daylilies during HAD. The results demonstrated that pretreatment was a critical step in regulating the drying quality of daylilies. Although conventional thermal pretreatments (HWB and SB) effectively inactivated enzymes but still caused color and flavor deterioration, while RT showed limited advantages beyond colchicine reduction. Although UT significantly accelerated drying and maintained microstructure, its excessive chlorophyll retention led to an undesirable greenish color, which fails to meet the commercial standard of golden-yellow daylilies. In contrast, CP exhibited significant advantages across multiple quality indicators, which were reflected in its optimal maintenance of color parameters and pigment composition, effective simultaneous inhibition of browning and 5-HMF accumulation, better retention of polyphenols, and the optimization of flavor formation pathways through chemical regulatory mechanisms. This study revealed the mechanisms of different pretreatments at multiple scales, providing a reliable scientific basis for the targeted process development of dried daylily products.

## Data Availability

The original contributions presented in the study are included in the article/supplementary material, further inquiries can be directed to the corresponding author.
